# IL-33 and Soluble ST2 Are Associated With Recurrent Spontaneous Abortion in Early Pregnancy

**DOI:** 10.3389/fphys.2021.789829

**Published:** 2022-01-13

**Authors:** Long Zhao, Jinhua Fu, Feng Ding, Juan Liu, Lin Li, Qi Song, Yinghui Fu

**Affiliations:** ^1^Department of Nephrology, The Affiliated Hospital of Qingdao University, Qingdao, China; ^2^Department of Obstetrics, Qingdao Jinhua Gynecology Hospital, Qingdao, China

**Keywords:** recurrent spontaneous abortion, IL-33, ST2, biomarker, diagnosis

## Abstract

Normal pregnancy is related to the successful transition from type 1 cellular immunity to type 2 cellular immunity. Therefore, this study aimed to investigate whether there is abnormal expression of cytokines in the process of inducing Recurrent spontaneous abortion (RSA). Interleukin (IL)-33 is a new member of the IL-1 family, and ST2, as IL-33’s receptor, induced the production of type 2 cytokines. In this study, blood samples were collected from 19 non-pregnant women of normal childbearing age, 28 normal pregnant women, and 33 women with RSA. The serum concentrations of IL-33 and ST2 were detected by flow cytometry. Our results showed that the serum concentrations of IL-33 and ST2 in the RSA group were significantly higher than those in the healthy control group (IL-33: *P* < 0.05; ST2: *P* < 0.0001), and IL-33 and ST2 had a higher level in the process of RSA predictive value. In addition, this study initially found that the serum concentrations of IL-33 and ST2 were not significantly correlated with the number of weeks of pregnancy, and there was a lower correlation between IL-33 and ST2 during RSA. This result may be related to the small number of cases. This study is the first time to correlate the changes in serum concentrations of IL-33 and ST2 with RSA, which may be a novel biomarker for the prediction and treatment of RSA.

## Introduction

In China, the occurrence of ≥ 2 consecutive fetal losses is defined as recurrent spontaneous abortion (RSA), a serious reproductive disorder of pregnancy with a prevalence as high as 1 to 5% in women of reproductive age (Practice Committee of the American Society for Reproductive Medicine, 2012). Recent studies have shown that the etiology of RSA is complex and varied, including advanced age, genetic abnormalities, infections, immune disorders, coagulation abnormalities, and endocrine dysfunction (Guo et al., 2020). However, the etiology of most RSA is unknown. Interestingly, more than half of the cases in which RSA occurs present with immune dysfunction (Baek et al., 2007), and these cases are often referred to as immune-related RSA (Alijotas-Reig and Garrido-Gimenez, 2013). Therefore, understanding the early manifestations and mechanisms of RSA will help us to take early steps to prevent pregnancy failure.

The success of pregnancy is based on the completion of an accurate switch of immune defense mechanisms during the early stages of embryo colonization (Wang et al., 2020). The embryo is a semi-allogeneic graft to the mother in early gestation and is therefore antigenic in its initial stages of development. The uterus, as the site of embryo implantation and development, is non-specific in its initial immune response to the embryo. The endometrial stroma contains a large number of immune cells, including NK cells, innate lymphocytes, macrophages, decidual dendritic cells and T cells (Tian et al., 1998). These cells form a pro-inflammatory microenvironment by producing a range of cytokines and chemokines that promote embryonic attachment (Dimitriadis et al., 2005). Subsequently, the Th1-type immune response rapidly changes to a Th2-type immune response, which primarily controls the endocrine and immune response and allows the pregnant woman to continue a safe pregnancy. Available evidence suggests that disturbances in the endometrial immune microenvironment are associated with repeated implantation failure (RIF) and unexplained RSA (Liu et al., 2019). We therefore hypothesize that the cause of RSA may be related to the unsuccessful conversion of Th1 to Th2 immune responses.

Growth stimulation expressed gene 2 (ST2) protein is a member of the interleukin 1 receptor/Toll-like receptor superfamily with two forms, i.e., soluble ST2 (sST2) and *trans*-model ST2 (ST2L). ST2 was previously considered as a key protein affecting cell proliferation and has an impact on tumor development (Werenskiold et al., 1989). ST2L can be expressed in a variety of cells, such as mast cells, macrophages and some lymphocytes (NK cells and Th2 cells, etc.) (Klemenz et al., 1989; Kumar et al., 1997; Xu et al., 1998; Kuroiwa et al., 2001; Allakhverdi et al., 2007; Smithgall et al., 2008), and it is highly expressed in immune cells that mediate Th2-type immune responses. All these studies suggest that ST2 is inextricably linked to the Th2-type immune response, however, it is unknown whether ST2 plays a role in RSA associated with abnormal Th2-type immune response. Furthermore, as ST2 becomes more widely used, it has been found to be associated with lung disease, sepsis and gastrointestinal disorders (Boga et al., 2016) and is becoming one of the important markers in the management of heart failure to indicate disease progression and prognosis (Daniels and Bayes-Genis, 2014).

Interleukin (IL)-33 is an important ligand for ST2. IL-33 is a dual-function cytokine whose full-length form acts as a transcription factor in the nucleus and inhibits the expression of NF-κB, a gene that regulates pro-inflammatory signaling. When cells are subjected to external stimuli or cellular necrosis, the precursor of IL-33 can be processed by neutrophil-derived proteases into the mature IL-33 form, which is then released as a cytokine into the extracellular compartment (Moussion et al., 2008). Mature IL-33 can bind to ST2L and trigger an inflammatory cascade response. However, sST2 binding to IL-33 prevents its binding to ST2L in immune cells, which in turn inhibits the activation of Th2 cells and the release of anti-inflammatory cytokines (IL-4, IL-5, IL-10, IL-13), and enhances the activation of Th1 cells and the release of pro-inflammatory cytokines (TNF-α, etc.) (Dinarello et al., 2012). Thus, IL-33/ST2 has an immunomodulatory role and this signaling axis may play an important role in a variety of diseases associated with major Th2 immune responses, such as rheumatoid arthritis, sepsis, and ulcerative colitis (Moussion et al., 2008; Dinarello et al., 2012; Daniels and Bayes-Genis, 2014; Pei et al., 2014; Boga et al., 2016).

In summary, we propose the following hypothesis: IL-33 and sST2 in RSA patients may contribute to RSA by attenuating the conversion to Th2-type immune responses. We plan to examine serum levels of IL-33 and sST2 in women with a history of RSA and normal women in the unpregnant and pregnant states to discover they whether are consistent with our hypothesis or have some diagnostic value.

## Materials and Methods

### General Data

Eighty patients who attended Qingdao Jinhua Gynecological Hospital between December 2019 and December 2020 were selected for retrospective analysis, 19 normal non-pregnant women, 28 normal pregnant women, six non-pregnant women with a history of RSA and 27 pregnant women with a history of RSA. Diagnostic criteria for RSA: referring to the diagnostic criteria of the American Society for Reproductive Medicine, ≥ 2 consecutive losses of pregnancies was defined as RSA.

Inclusion criteria for the normal group: (i) no history of miscarriage; (ii) age ≥ 20 years and ≤ 40 years; (iii) no uterine abnormalities detected by hysteroscopy or ultrasound examination of the uterus and both adnexa; (iv) normal karyotype of both spouses.

Inclusion criteria for RSA group: ① diagnosis of RSA; ② age ≥ 20 years, ≤ 40 years; ③ no uterine abnormalities were found by hysteroscopy or uterine and bilateral adnexal ultrasonography; ④ normal karyotype of both spouses.

Exclusion criteria for the normal and RSA groups: ① abnormal reproductive endocrine hormones: diabetes mellitus, thyroid disease, etc. before pregnancy; ② suffering from infectious diseases; ③ cases with incomplete data.

### Main Reagents and Equipment

Human ST2/IL-1R4 Kit and Human IL-33 Assay Kit were purchased from Beijing QuantiBio Biotechnology Co., Ltd. Plate washer (Beijing QuantoBio Biotechnology Co., Ltd.). MS3 digital display oscillator (IKA, Germany). Centrifuge (Thermo Scientific). Flow cytometer BeamCyte-1026 (Changzhou BeamDiag Biotechnology Co., Ltd.).

### Assay Method

The serum of 19 normal non-pregnant women, 28 normal pregnant women at 7–19 weeks of gestation, and 33 women with a history of RSA (6 non-pregnant women and 27 pregnant women at 7–30 weeks of gestation) were collected in 1 mL each. The reacted samples were fluorescently detected by the flow cytometer equipped with a 488 nm laser excited PE channel in combination with the standard curve of antigen standards for the quantitative detection of sST2 and IL-33.

(1) Take a 96-well filter plate. Add 20 μL of 1 × capture microsphere mixture to each well (vortex for 45 s before adding the microspheres).
(2) Add 20 μL of gradient diluted standards to wells #1-8, 20 μL of experimental diluent to well #9 as the background well, and 20 μL of sample in each of the other wells.
(3) Add 20 μL of 1 × detection antibody mixture to each well and incubate at room temperature for 2 h with shaking frequency of 500 rpm, protected from light.
(4) Add 20 μL of SA-PE to each well and incubate at room temperature for 30 min with shaking frequency of 500 rpm.
(5) Add 200 μL of 1 × buffer to each well and remove the liquid from the well with a strainer. Then repeat once.
(6) Add 100 ∼ 200 μL/well of 1 × buffer and detect.

### Statistical Methods

The statistical and graphing software was GraphPad Prism 8. The measurement data were expressed as mean ± standard error (Mean ± SEM), and the comparison of IL-33 and sST2 indexes between the two groups was done by group *t*-test, and the ROC curve was plotted to assess the predictive value of IL-33 and sST2 serum concentration in RSA by Area Under Curve (AUC). The difference in the predictive value of IL-33 and sST2 serum concentrations in RSA was considered statistically significant at *P* < 0.05.

## Results

### Serum IL-33 and sST2 Levels in Pregnancy

In this study, we first assessed the serum concentrations of IL-33 and sST2 in pregnant women during pregnancy and collected and analyzed the levels of IL-33 and sST2 in the normal group (non-pregnant, pregnant) and the diseased group with a history of RSA (non-pregnant, pregnant), respectively. The basic information about the cases were shown in Supplementary Table 1.

**FIGURE 1:**
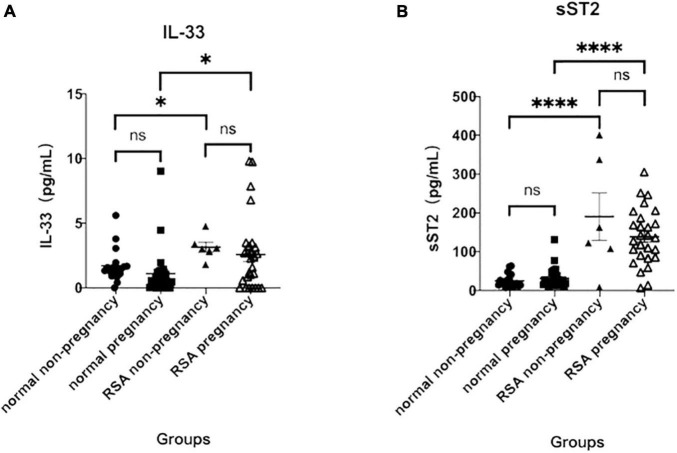
Serum IL-33 and ST2 levels at 7–19 weeks of gestation. **(A)** Analysis of serum IL- 33 in the normal non-pregnancy, normal pregnancy, RSA non-pregnancy and RSA pregnancy groups. **(B)** Analysis of soluble ST2 in the same groups. (^∗^*p* < 0.05 versus normal pregnancy group; ^∗∗∗∗^*p* < 0.0001 versus normal pregnancy group).

In the normal group, serum IL-33 levels were 1.10 ± 0.34 (pg/mL) in those with 7–19 weeks of gestation (median 12 weeks) and 1.71 ± 0.29 (pg/mL) in the non-pregnant group, which were not statistically different from those in the pregnant group. In the diseased group, serum IL-33 levels were 3.15 ± 0.39 (pg/mL) in the non-pregnant and 2.58 ± 0.55 (pg/mL) in those at 7–30 weeks of gestation (median 11 weeks), which were not statistically different from each other and were significantly higher than those in the normal group, with statistically significant differences (*P* < 0.05) (Figure 1A).

Similar to IL-33 levels, serum levels of sST2 were not statistically different between pregnant [31.57 ± 4.83 (pg/mL)] and non-pregnant [25.10 ± 3.88 (pg/mL)] in the normal group. In the RSA group, sST2 was similar in the pregnant [138.70 ± 14.04 (pg/mL)] and in the non-pregnant [190.40 ± 60.90 (pg/mL)] and was significantly higher than in the normal group (*P* < 0.0001), and all differences were statistically significant (Figure 1B). The results suggest that elevated serum IL-33 and sST2 levels may be associated to RSA.

### Predictive Value of Maternal Serum IL-33 and ST2 on Recurrent Spontaneous Abortion

To clarify the predictive value of serum IL-33 and ST2 levels on RSA, we performed ROC curve analysis of serum IL-33 and sST2 levels between groups.

**FIGURE 2:**
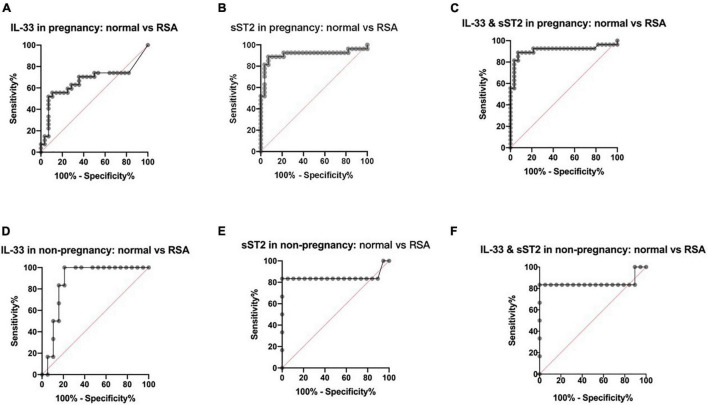
Predictive value of maternal serum IL-33 and ST2 level in RSA. **(A)** ROC curve analysis of serum IL-33 level in the pregnancy groups, AUC=0.6660. **(B)** ROC curve analysis of serum solubleST2 level in the pregnancy groups, AUC=0.9087. **(C)** ROC curve analysis of serum IL-33 and sST2 levels in the pregnancy groups, AUC = 0.9107. **(D)** ROC curve analysis of serum IL-33 level in the non-pregnancy groups, AUC=0.8684. **(E)** ROC curve analysis of serum solubleST2 level in the non- pregnancy groups, AUC=0.8465. **(F)** ROC curve analysis of serum IL-33 and sST2 levels in the non-pregnancy groups, AUC=0.8509.

The results showed that for those who were pregnant, the area under curve (AUC) of IL-33 in the disease versus normal groups was 0.6660 (Figure 2A), with a 95% confidence interval (CI) of 0.5120–0.8200 (*P* < 0.05), and the AUC of sST2 was 0.9087, with a 95% CI of 0.8139–1.000 (*P* < 0.0001) (Figure 2B), and the AUC for the combination of both was 0.9107, with a 95% CI of 0.8172–1.000 (*P* < 0.0001) (Figure 2C), indicating that the combination of serum IL-33 and sST2 levels has a similar high predictive value for RSA after pregnancy with the serum sST2 level.

For those who were not pregnant, the AUC of IL-33 was 0.8684, with a 95% CI of 0.7271–1.0000 (*P* < 0.01) in the RSA group versus the normal group (Figure 2D), while the AUC of sST2 was 0.8465, with a 95% CI of 0.5705–1.000 (*P* < 0.05) (Figure 2E), and the AUC of the combination of the two was 0.8509, with a 95% CI of 0.5823–1.000 (*P* < 0.05) (Figure 2F), indicating that serum IL-33 and sST2 levels have some predictive value for RSA before pregnancy.

### Relationship Between Maternal Serum IL-33 and sST2 and Gestational Age or Age

**FIGURE 3:**
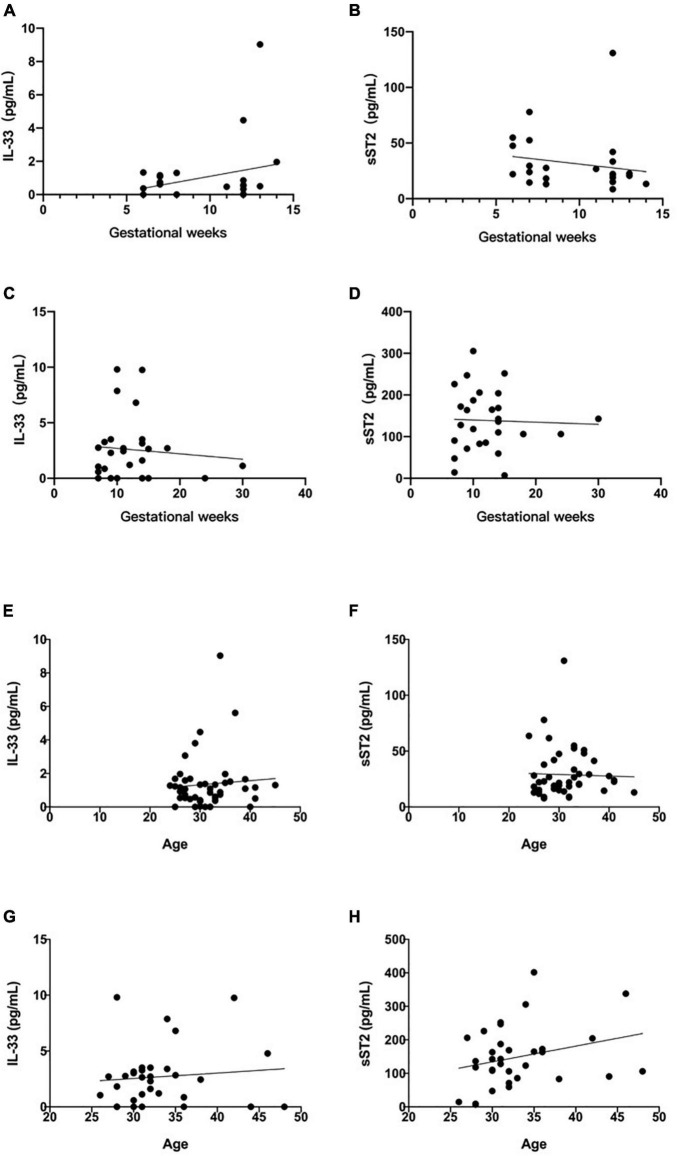
Relationship between maternal serum IL-33 and sST2 level and gestational age. **(A)** Correlation between serum IL-33 and weeks of gestation in normal pregnancy group. **(B)** Correlation of soluble ST2 with the number of weeks of gestation in normal pregnancy group. (Values corresponding to the same number of weeks of gestation are plotted using their mean values). Relationship between maternal serum IL-33 and ST2 level and gestational weeks. **(C)** Correlation between serum IL-33 and weeks of gestation in RSA pregnancy group. **(D)** Correlation of soluble ST2 with the weeks of gestation in RSA pregnancy group. (Values corresponding to the same number of weeks of gestation are plotted using their mean values). Relationship between maternal serum IL-33 and ST2 level and age. **(E)** Correlation between serum IL-33 and age in normal group. **(F)** Correlation between serum soluble ST2 and age in normal group. Relationship between maternal serum IL-33 and ST2 level and age. **(G)** Correlation between serum IL-33 and age in diseased group. **(H)** Correlation between serum soluble ST2 and age in diseased group.

To further clarify the predictive value of IL-33 and sST2 for RSA, we statistically analyzed whether the elevated serum IL-33 and sST2 were related to gestational age, respectively. The results showed that there was no significant correlation between serum IL-33 and sST2 levels and gestational age in the normal group (IL-33: *R*^2^ = 0.0682, *P* = 0.2072. sST2: *R*^2^ = 0.0309, *P* = 0.4007, Figures 3A,B), and no significant correlation between serum IL-33 and sST2 levels and gestational age in the diseased group (IL-33: *R*^2^ = 0.0080, *P* = 0.6565. sST2: *R*^2^ = 0.0014, *P* = 0.8531, Figures 3C,D). However, in the present study, both IL-33 and ST2 serum concentrations peaked at week 11–12, but whether this is statistically significant needs to be further verified with a larger sample size.

To age, the results showed that there was no significant correlation between them in the normal group (IL-33: *R*^2^ = 0.0060, *P* = 0.6039. sST2: *R*^2^ = 0.0010, *P* = 0.8342, Figures 3E,F), and no significant correlation between them in the diseased group (IL-33: *R*^2^ = 0.0099, *P* = 0.5820. sST2: *R*^2^ = 0.0775, *P* = 0.1168, Figures 3G,H).

### Relationship Between Maternal Serum IL-33 and sST2 and Number of Miscarriage

**FIGURE 4:**
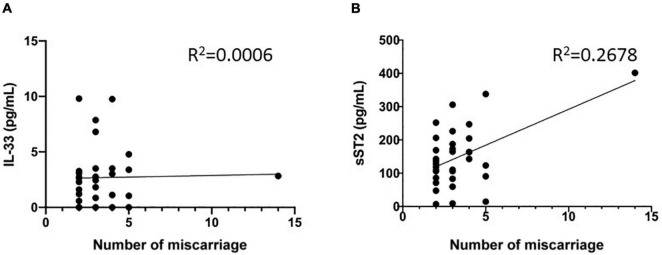
Relationship between maternal serum IL-33 and ST2 level and number of miscarriage. **(A)** Correlation between serum IL-33 and number of miscarriage. **(B)** Correlation between serum soluble ST2 and number of miscarriage.

We also statistically analyzed whether the elevated serum IL-33 and sST2 were related to number of miscarriage in diseased group. The results showed that there was no correlation between the serum IL-33 level and the number (*R*^2^ = 0.0006, *P* = 0.8934, Figure 4A). However, there was significant correlation between the sST2 levels and the number of miscarriage (*R*^2^ = 0.2678, *P* = 0.0020, Figure 4B). This result supports that sST2 levels are predictive for RSA.

### Correlation of Maternal Serum IL-33 and sST2

**FIGURE 5:**
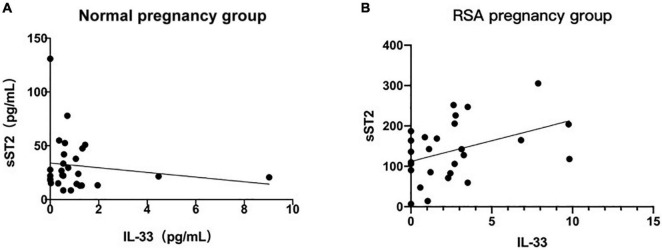
Correlation of maternal serum IL-33 and ST2 level. **(A)** Correlation analysis between serum IL-33 and ST2 level in the normal pregnancy group. **(B)** Correlation between serum IL-33 and ST2 level in the RSA pregnancy group. (Values corresponding to the same number of weeks of gestation are plotted using their mean values).

In the above study, the serum concentrations of IL-33 and sST2 in the RSA group alone suggested a high predictive value, but further studies are needed to determine whether the combination of these two indicators can improve the diagnosis of RSA. The results showed that the serum concentrations of IL-33 and sST2 did not correlate in normal pregnancies (*R*^2^ = 0.0231, *P* = 0.4401), and their correlation coefficients were significantly higher in RSA than in normal pregnancies, although they still did not correlate significantly in the RSA group (*R*^2^ = 0.1616, *P* < 0.05) (Figures 5A,B). Thus, we speculate that IL-33 in serum of patients with RSA may be correlated with sST2, and the negative results of this study may be related to the small sample size.

## Discussion

IL-33 is a member of the IL-1 cytokine family and is involved in both the innate and adaptive immune responses through interaction with its receptor ST2. Activation of IL-33/ST2 signaling activates pleiotropic immune functions in a variety of immune cells that can express ST2 protein, including macrophages, neutrophils, eosinophils, basophils, mast cells, Th2 cells, regulatory T (Treg) cells, etc. (Griesenauer and Paczesny, 2017). Recently, it has been shown that IL-33 is involved in the pathogenesis of several inflammatory diseases, including allergic diseases (Liew et al., 2016; Gupta et al., 2017), infectious diseases (Donovan et al., 2016; Xu et al., 2017), and neuropathic pain (Fattori et al., 2017). In addition, sST2 levels are significantly elevated in patients with heart disease and graft-versus-host disease (Mathews et al., 2016; Hartwell et al., 2017). Therefore, IL-33 and its receptor ST2 may be promising new targets for the treatment of RSA.

In this study, patients with RSA were collected as the main study population. Given that RSA is a type of pregnancy loss that may be caused by a failure of transformation of the immune response, we hypothesized that there may be abnormalities in the indicators associated with Th2-type immune transformation during this process. The present study showed a significant increase in serum IL-33 and sST2 in the RSA group compared to the normal pregnancy group (Figure 1), which further validates our hypothesis that RSA is accompanied by abnormal Th2-type immune transformation. Granne et al. (2011) previously reported a significant increase in ST2 expression in patients with preeclampsia, and this increase was detectable prior to the onset of clinical symptoms. During pregnancy, sST2 production may reflect the enhanced Th1-type inflammatory environment of the disease compared to normal pregnancy, as sST2 acts as a decoy receptor for IL-33, competing with membrane-bound ST2L. Thus, the very high circulating sST2 levels seen in preeclampsia would be predicted to reduce the binding of IL-33 to its receptor, thus contributing to the Th1-type bias seen in this disorder. This finding has some similarity to the present study, both suggesting that abnormalities in immune transformation may accompany the abnormal course of pregnancy. In addition, this study also found small amounts of IL-33 and sST2 expression in the normal non-pregnant group and in the normal pregnancy group, and serum sST2 levels were much higher in the RSA group than in the healthy controls (*p* < 0.0001). It was found that ST2 protein was expressed in alveolar epithelial cells, cardiomyocytes, and also in brain and small intestine cells (Homsak and Gruson, 2020), so the placenta may not be the only source of high levels of sST2 in the serum of patients with RSA.

In this study, serum IL-33 and sST2 were found to have predictive value for RSA for the first time (Figure 2), however, there was no significant correlation between IL-33 and sST2 and gestational age or age in both the normal and RSA groups (Figure 3). Combined with the analysis of Granne et al. (2011), the lower correlation in this study may be related to the small sample size, which does not negate the predictive value of IL-33 and sST2 in RSA.

Further analyzing the relationship between IL-33 and sST2 levels and the number of miscarriage that had occurred in the disease group of patients, we also found that sST2 levels appeared to be higher in patients with a greater number of miscarriages. Given the insufficient sample size, only one patient with a high number of miscarriages seems to be the main factor that enhances the association (Figure 4B), more samples and more rigorous experiments are needed to prove or falsify this hypothesis.

In our clinical case collection work, we found that pregnant women with RSA often have some endocrine disorders, such as diabetes mellitus and thyroid disease. Diabetes mellitus usually causes microangiopathy, which in turn leads to chronic progressive lesions, hypofunction and failure of tissues and organs such as eyes, kidneys, nerves, heart and blood vessels. It has been shown that the IL-33/ST2 signaling pathway is activated in diabetic nephropathy (Elsherbiny et al., 2020), and endothelial dysfunction can be induced during the development of diabetic nephropathy (Xue et al., 2020). Interestingly, ST2, the key protein in this study, can be produced by endothelial cells, which activate immune responses and trigger organismal protective mechanisms by expressing large amounts of ST2 during the sepsis process (Huang et al., 2020). Thus, we speculate whether the high secretion of ST2 by endothelial cells is another influencing factor in the elevation of ST2 in the sera of pregnant women with RSA with diabetes? However, this influencing factor has not been addressed in the present study, and we will continue to investigate this issue in subsequent studies as well.

Since we observed a significant increase in both IL-33 and sST2 in patients with RSA, this phenomenon may be due to the maternal immune system’s response to preeclampsia. In contrast, the elevated serum sST2 may also be associated with the underlying maternal disease itself. Ultimately the maternal incomplete or inadequate conversion of the Th2-type immune response is due to the net effect of increased IL-33 and sST2 levels. It is a pity that the correlation results between IL-33 and sST2 were not obtained in this study, which we will further investigate in our future work.

In conclusion, in this study, IL-33 and sST2 were found to be significantly increased in patients with recurrent miscarriage for the first time. IL-33 and sST2 may become novel biomarkers for early prediction of pregnancy failure and recurrent miscarriage, providing potential new targets for the treatment of RSA.

## Data Availability Statement

The raw data supporting the conclusion of this article will be made available by the authors, without undue reservation.

## Ethics Statement

Ethical review and approval was not required for the study on human participants in accordance with the local legislation and institutional requirements. The patients/participants provided their written informed consent to participate in this study.

## Author Contributions

LZ, JF, FD, and JL: conception and design, data analysis and interpretation. LZ and JF: administrative support. FD, JL, LL, QS, and YF: provision of study materials or patients, collection and assembly of the data. All authors wrote the manuscript and approved the final version of the manuscript.

## Conflict of Interest

The authors declare that the research was conducted in the absence of any commercial or financial relationships that could be construed as a potential conflict of interest.

## Publisher’s Note

All claims expressed in this article are solely those of the authors and do not necessarily represent those of their affiliated organizations, or those of the publisher, the editors and the reviewers. Any product that may be evaluated in this article, or claim that may be made by its manufacturer, is not guaranteed or endorsed by the publisher.
